# Study on emergency evacuation in underground urban complexes

**DOI:** 10.1371/journal.pone.0278521

**Published:** 2022-12-30

**Authors:** Junjie Wei, Qigen Deng, Lixin Zhang

**Affiliations:** 1 School of Safety Science and Engineering, Henan Polytechnic University, Jiaozuo, China; 2 Key Laboratory of Public Security Management Technology, Shandong Management University, Jinan, China; 3 Sichuan Xintairan Safety Technology Co., LTD, Chengdu, China; 4 College of Engineering, China University of Geosciences (Wuhan), Wuhan, China; Rutgers University, UNITED STATES

## Abstract

With progressive urbanization and the development and utilization of urban underground space, underground urban complexes (UUCs) have been increasingly used. UUCs have brought much convenience to people’s lives. However, due to their enclosed nature and complexity, it has been an urgent issue to avoid (or reduce) casualties and allow rapid and safe evacuation of people during an emergency. In this study, the evacuation simulation software Pathfinder was used. Based on the steering model, the variation of the total evacuation time and pedestrian flow at main exits with different simulated evacuation measures, congestion at key nodes and people’s path selection were compared and analyzed. Then, the critical locations in the spatial layout of UUCs that were prone to evacuation bottleneck effects were focused on and determined. The evacuation effectiveness of UUCs in an emergency was studied to investigate the problems of emergency evacuation in UUCs. It is found that in UUCs, the bottleneck effects were likely to occur at stairway entrances and exits as well as supermarket checkout counters and caused severe congestion. These locations should be focused on during emergency evacuation. For key locations prone to evacuation bottlenecks, increasing the width of exits or setting up auxiliary evacuation channels could be an effective measure to improve evacuation efficiency. In addition, formulating rational evacuation rules can be a favorable measure for emergency evacuation. However, during the evacuation, the herd mentality in people has an uncertain (positive or negative) impact on evacuation effectiveness. Setting up diversion walls may improve evacuation efficiency and reduce congestion to a certain extent, while evacuation confusion and chaos are prone to occur after diversion. These findings in this study have significant implications for improving the emergency management of UUCs.

## 1. Introduction

The accelerated urbanization process has exacerbated the contradiction between limited land resources and the demand for construction land [1]. In order to alleviate this contradiction and to make people’s productive life more convenient, single-function residential, industrial and commercial areas have developed into a new form of urban organization integrating housing, entertainment and business—the urban complex [2, 3]. The complex indicates a highly dense open public space formed by combining multifunctional buildings in a group of limited attributes [4]. When the surface space in large urban centers becomes overly dense, commercial streets and parking lots are relocated underground, gradually forming a new type of mixed-use building—the underground urban complex (UUC) [5, 6]. UUCs have become a major urban construction and renewal form, and construction investments are significantly increasing [7–9]. The development of UUCs and their inclusion of multiple urban functions induce more complex internal structures, highly enclosed spaces, fewer entrances and exits, and a dense population during operation [10]. When emergencies occur, people can only escape through narrow escape routes such as stairwells or evacuation routes. The evacuation capacity of UUCs is significantly reduced compared with that of above-ground urban complexes [11]. People in UUCs are more likely to show tension and panic [12]. It is widely reported that casualties in UUCs are caused by people failing to evacuate safely and effectively during emergencies [13–15].

The evacuation process is a key part of the emergency response strategy to manage and control various emergencies or disasters. Evacuation studies of evacuees can quantify their evacuation behavior and facilitate more scientific and rational evacuation designs [16, 17]. An important criterion for evacuation design is the ability to evacuate people to a safe area before danger strikes while evacuating people from hazardous urban complexes has always been a major and challenging task [18]. Currently, there are more studies on the safe emergency evacuation of single buildings or complexes above ground [19–27], while fewer studies were focused on the safe evacuation of UUCs compared to above-ground buildings. Existing research methods for emergency evacuation include accident investigation, controlled experiments, numerical modeling and crowding experiments based on the effect of the inner emotions of animals on evacuation behavior [28–31]. Numerical modeling can save costs and better reflect the real evacuation environment by developing people movement rules and has been increasingly applied to emergency evacuation research in recent years [13, 32, 33]. There are many research models for evacuation worldwide, with different classification forms according to different criteria, such as micro- and macro-models [34, 35], discrete and continuum models [36, 37], deterministic and stochastic models [38], rule-based [39–41] and force-based models [42], and high and low simulation models. The methods of evacuation simulation range from manual calculations to complex numerical modeling [17, 19, 43].

Some scholars have compared the evacuation results using the manual approach and Pathfinder numerical modeling. Research shows that numerical simulation can more intuitively reflect the evacuation process, the congestion and bottlenecks of safety exits and evacuation routes, and is more suitable for the optimal fire protection design in the evacuation of underground commercial buildings. Numerical modeling in evacuation studies allows for the analysis of multiple scenarios based on the needs and possible threats of the studied case, while the manual approach is more applicable to standardized scenarios [44, 45]. Numerical modeling allows overall evacuation performance evaluation using accurate and representative scenarios, thus achieving multi-parameter evaluation and management of problems. These cannot be achieved using only "manual" methods [46]. Presently, scholars have conducted some studies on the evacuation of subway stations [13, 46–52] and their findings have played a role in the emergency management of UUCs. It should be noted that evacuation for UUCs has started to attract academic attention. Based on the system dynamics theory, Li et al. [10] established a simplified model for evacuating large-scale groups in UUCs. They studied the group evacuation behavior at a different initial number of evacuees. This model combined the advantages of continuous and discrete models, which provided better theoretical support for the safety management practice of UUCs. However, the evacuation of UUCs has been rarely studied, compared with that of ordinary above-ground buildings. Thus, further in-depth studies are needed.

With the advancement of urbanization in China, the construction of UUCs has been developing rapidly. The development and utilization of UUCs have increasingly extended to deeper spaces and become more complicated. This brings about a challenge to an emergency evacuation. Therefore, it is important to conduct the evacuation simulation study of UUCs to improve evacuation efficiency and guarantee the safety of people’s lives and property. This study focused on the critical locations prone to evacuation bottleneck effects in the spatial layout of UUCs. A simulated evacuation model under different evacuation measures was constructed using Pathfinder. The effects of different evacuation measures on total evacuation time and pedestrian flow at main exits, as well as the effects of congestion at key nodes and selection of pedestrian flow paths on the evacuation of UUCs during emergencies, were compared and analyzed. The "GG" UUC in Wuhan, China (with common characteristics of UUCs) was selected as a representative for UUC modeling in order to obtain effective evacuation measures for the evacuation process of UUCs and provide some support for emergency management. This study can provide a basis for the scientific formulation of safety management strategies for UUCs in the future and improve the reliability, self-adaptability and efficiency of safety management systems for UUCs.

## 2. Methods

### 2.1 Pathfinder

In this study, Pathfinder, developed by Thunderhead engineering, Inc., U.S., was used to conduct simulation analysis. Pathfinder can achieve intelligent body-based continuum evacuation, agent-based evacuation and human movement simulation. This software includes three modules: graphical interface, simulator and result display program [13]. The result display program includes 2D floor plans, 3D evacuation animations and a summary text file. People’s movement patterns include Society of Fire Protection Engineers (SFPE) mode and Steering mode [53, 54]. SFPE behavior is the most basic behavior. Flow-based selection means people will automatically move to the nearest exit and will not interact with each other, but the queue will conform to SFPE assumptions. This mode uses spatial density to determine people’s movement speed based on SFPE Fire Protection Handbook Protection Engineering and SFPE Engineering Guide—Human Behavior Fire Use.

The Steering mode uses a combination of path planning, guidance mechanisms and collision handling to control people’s movement. It can generate new paths to adapt to a new evacuation environment if the distance between people and the path to the nearest point exceeds a certain threshold. One of the Steering modes reflects people’s interaction, allowing a more realistic evacuation situation [55]. All simulation experiments in this paper used the Steering mode, where people’s movement was controlled by path planning, steering mechanisms and collision handling. Each evacuee followed a path connecting the current location and the target point, and this path controlled the entire route of the evacuation simulation. If the distance between the evacuee and the nearest point exceeds a predetermined threshold, the initially planned evacuation route is redesigned to accommodate the new state. The main factors involved in people’s movement included maximum speed and acceleration, steering mechanism and movement rules.

### 2.2 Evacuation simulation model

In the evacuation simulation experiments, the influence of the evacuation process of UUCs on the evacuation effectiveness was analyzed mainly in terms of total evacuation time, the flow rate at exits and evacuation bottleneck. A basic evacuation simulation model was developed to analyze the possible problems in the evacuation process through the Steering model of Pathfinder, in combination with the actual situation of Wuhan ‘GG’ UUC. Then, the evacuation simulation optimization model was established and the optimized evacuation simulation effectiveness was observed, as shown in Table 1. By comparing the simulation results of different measures, more reasonable and effective evacuation measures could be proposed.

**Table 1:** Evacuation simulation models under different evacuation measures.

Evacuation simulation model	Evacuation measures
**Basic evacuation simulation model**	Evacuation process without auxiliary evacuation facilities; no evacuation guidance
**Optimized evacuation simulation model**	(a) Set up auxiliary evacuation facilities: set up special channels and diversion walls at critical locations; (b) Formulate special evacuation guidance rules; (c) Regulate the density of people.

## 3. Model development

### 3.1 Practical overview of UUCs

**Fig 1:**
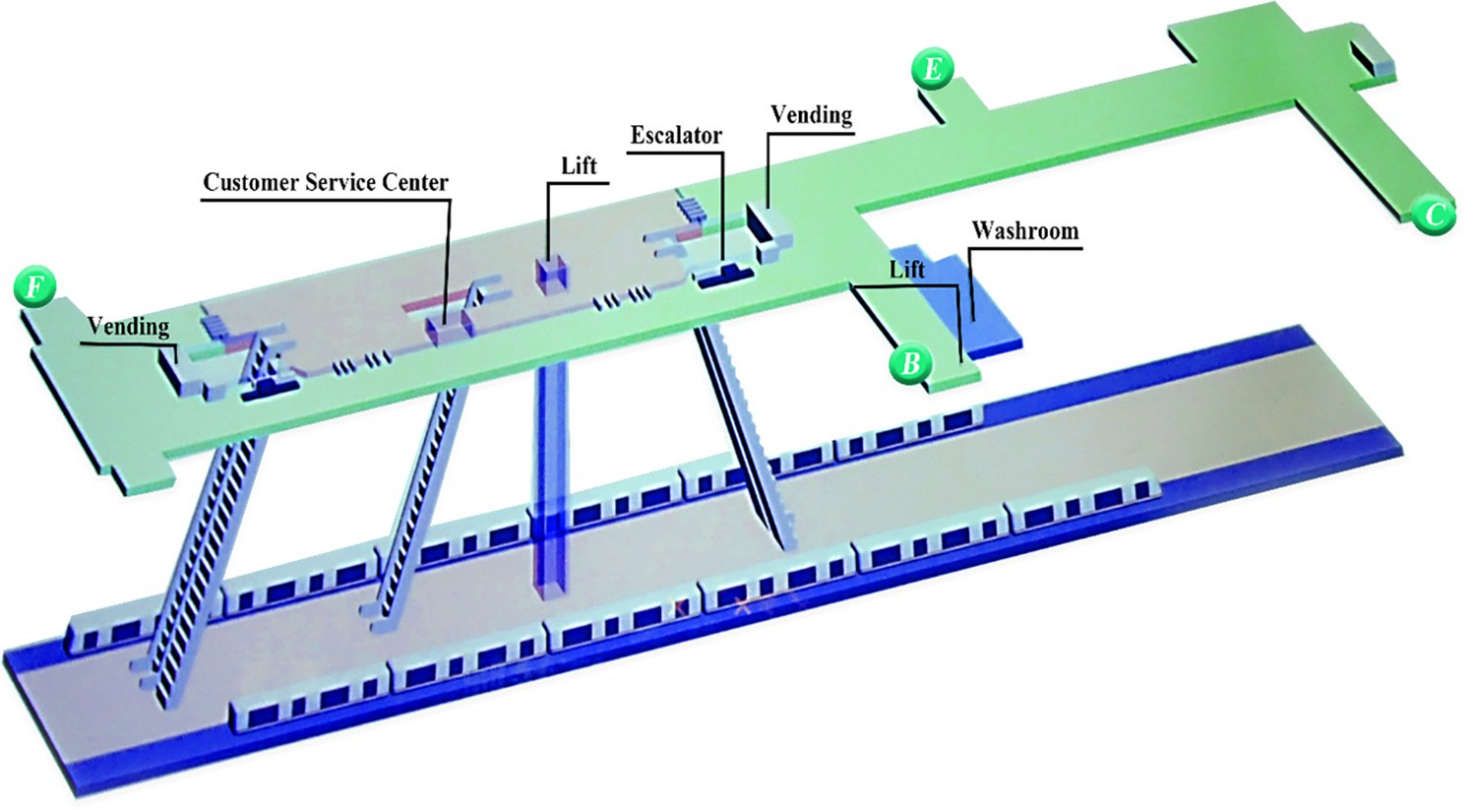
Partial 3D view of ‘GG’ UUC.

**Fig 2:**
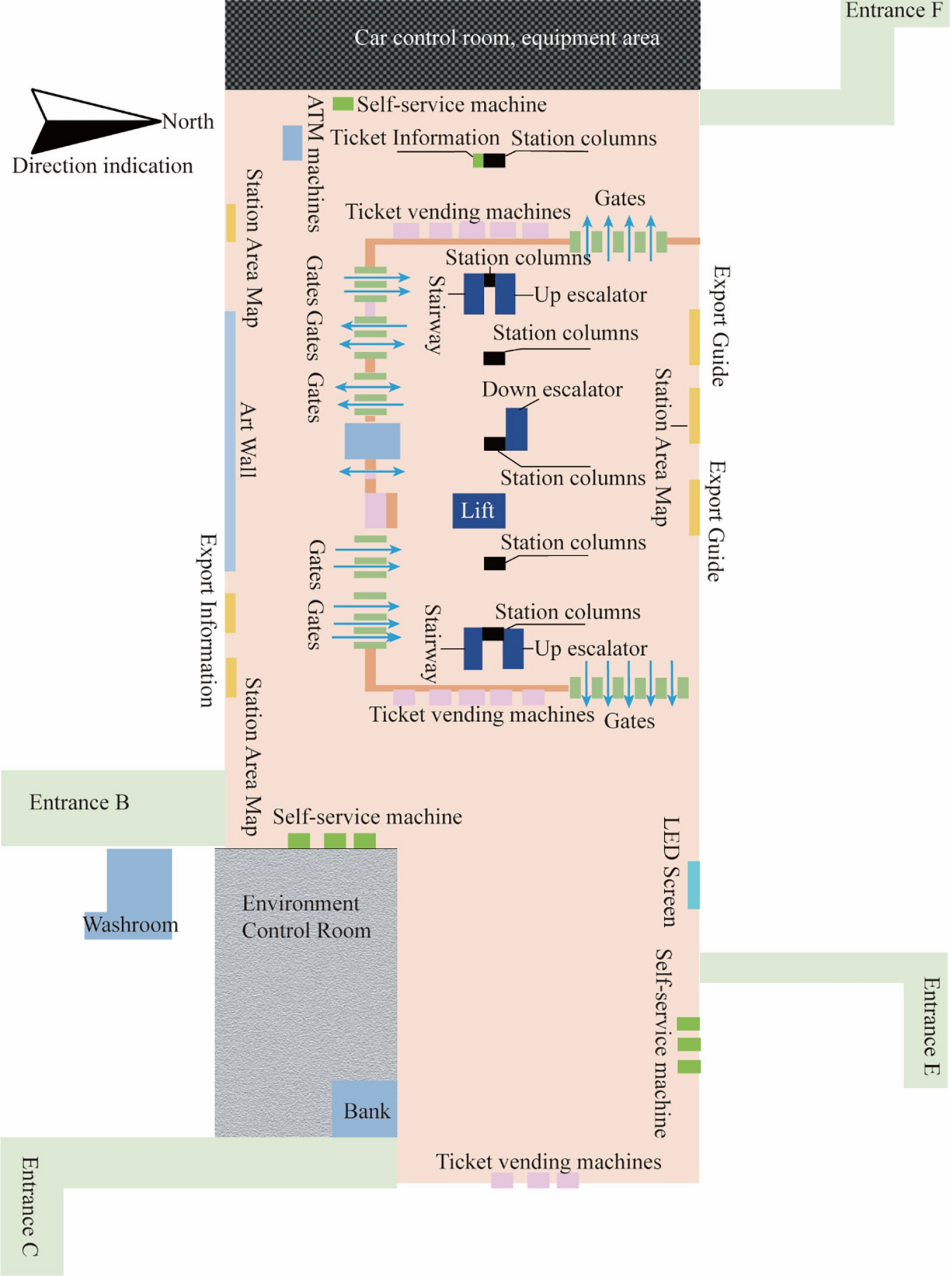
Subway station hall level plan.

**Fig 3:**
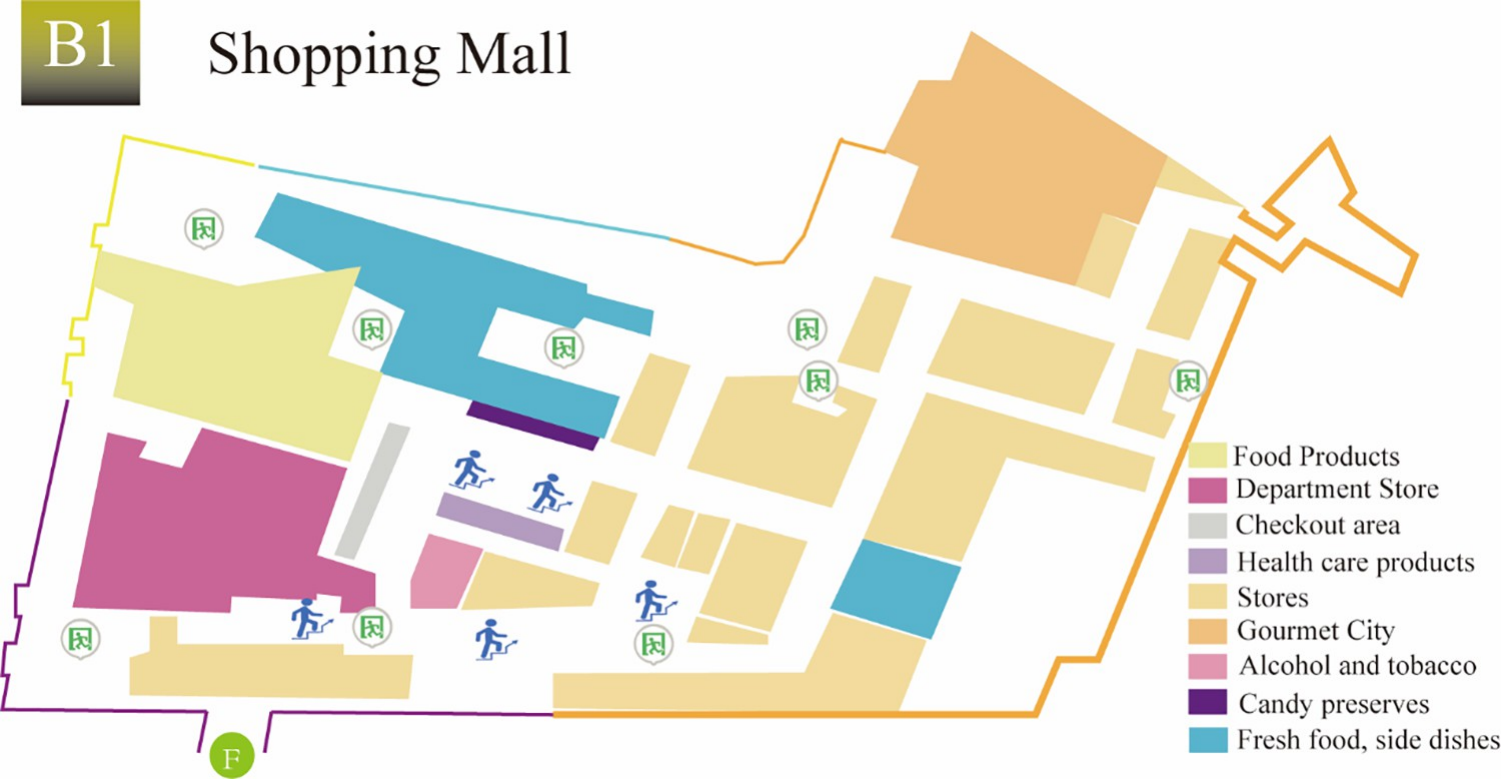
Shopping center B1 floor plan.

‘GG’ UUC is located in the urban planning area, which integrates social living spaces and municipal infrastructure spaces of different nature and uses such as transportation (subway station), shopping (shopping center), commerce, entertainment, leisure, parking and office. By introducing underground public spaces in horizontal and vertical directions, underground spaces of various functions are organized together in a comprehensive 3D manner. ‘GG’ UUC is a diverse, efficient, complex and unified building group. The subway station is a two-story underground island station. The partial 3D view of ‘GG’ UUC is shown in Fig 1. The first and second basement levels are the station hall level (Fig 2) and the platform level, respectively. The station has a total length of 278.7 m and a total construction area of about 13021.4 m^2^, and includes four exits (B, C, E and F), among which Exit F is connected to the B1 level of the shopping center, as shown in Fig 3. The subway station can be reached through the basement level of the shopping center.

### 3.2 Setting and construction of key model parameters

Before the model was established, some model parameters and basic rules were set as follows according to the situation of the ‘GG’ UUC:

1. Based on the field survey, there are 4 turnstile systems, with 21 turnstile gates in total in the ‘GG’ UUC subway station, with two types of gate widths: 90 cm, "wide gate"; 55 cm, "narrow gate". The location of the gates is arranged according to the actual situation. During the emergency evacuation in this simulation experiment, all gates are released and used as exit gates.
2. The width of the escalator in the subway station is 1.2 m, which can accommodate two people standing at the same time. During the evacuation, the elevator is powered off and used as an ordinary stair. Some escalators can only go down, but all are powered off during evacuation and can be used as evacuation stairs.
3. All vertical lifts in the station are not considered because vertical elevators cannot be used as evacuation facilities in emergencies. In addition, the advantage of elevators is not obvious on low floors. Vertical lift elevators are not drawn in the modeling simulation and are not used for evacuation.
4. Distribution of people: a total of 15,000 people are included. 10,000 people are randomly distributed on the platform level and station hall level of the ‘GG’ UUC subway station, and 5,000 people are randomly distributed on the B1 level and ground level 1 of the shopping center. People’s walking speed is set to obey a uniform distribution, with a minimum speed of 0.80 m/s and a maximum speed of 1.35 m/s. People’s shoulder width obeys uniform distribution, with a minimum shoulder width of 0.46 m and a maximum shoulder width of 0.52 m [53, 56]. The default values of the Pathfinder steering model were used for the settings of other parameters.

**Fig 4:**
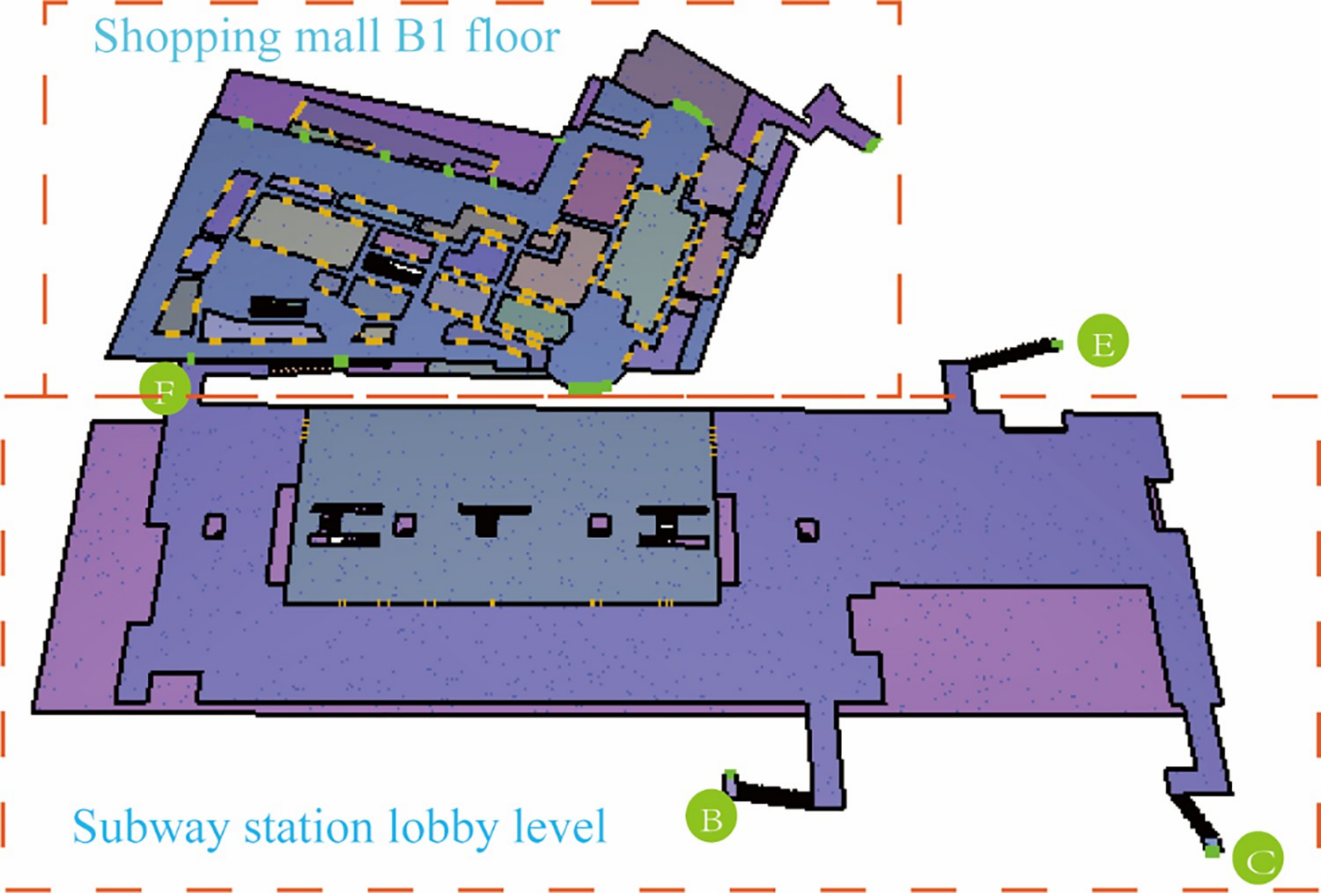
‘GG’ UUC model based on Pathfinder’s steering model.

According to the overview of the UUC in Section 3.1, the ‘GG’ UUC model was constructed based on Pathfinder, as shown in Fig 4.

## 4. Results and discussion

The basic evacuation simulation model was a simulation of evacuation without any auxiliary evacuation facilities and guidance rules. In the optimized evacuation simulation model, special passages were set up next to the gates in the basic evacuation simulation model. They were open so that the evacuees can also enter the station hall level. In addition, the optimized simulation model incorporated guidance rules, such as certain crowds taking Exit C to relieve the pressure on Exits B and E. The crowd was guided to reach the B1 level of the shopping center from Exit F, then take the stairs to the first floor and evacuate from the first-floor exit of the shopping center to the outdoors. Diversion walls were set up near Exits B and E to force people to line up so that some people stood at positions that did not interfere with evacuation. The queue was forced to wait for some time in these positions rather than flocking to all exits simultaneously.

**Fig 5:**
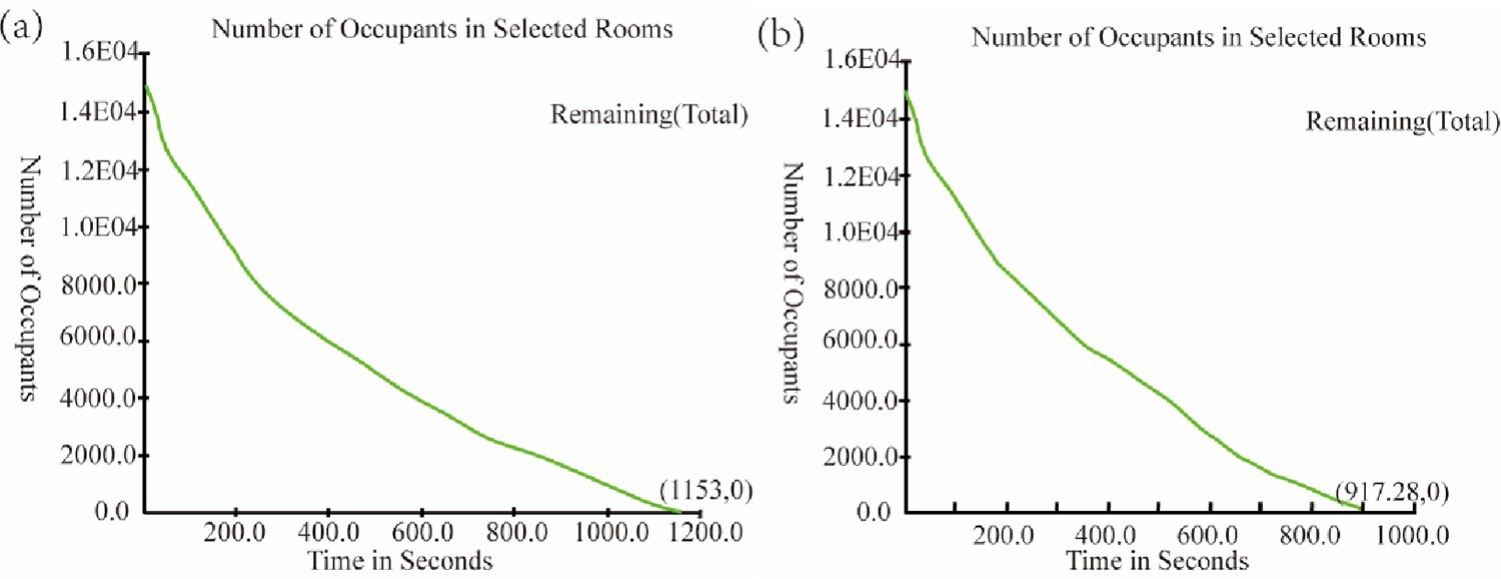
Curves of the total number of people remaining in the evacuation space during evacuation. (a) basic evacuation simulation model, (b) optimized evacuation simulation model.

The total number of people remaining in the evacuation space in the evacuation process of the basic evacuation simulation model and the optimized evacuation simulation model was analyzed. The curve of the total number with time is obtained, as shown in Fig 5(A) and 5(B). Fig 5(A) shows that in the basic evacuation simulation model, the remaining number of people in the evacuation space was 0 at 1153 s, i.e., the total evacuation time was 1153 s. The slope of the evacuation curve gradually decreased, indicating that the number of people evacuated to the outside safety area per unit time was large in the early evacuation period and gradually decreased with evacuation time. Fig 5(B) shows that in the optimized evacuation simulation model, the remaining number of people in the evacuation space was 0 at 917.28 s, i.e., the total evacuation time was 917.28 s. The time was 253.72 s smaller than that of the basic evacuation simulation model and the evacuation efficiency was improved by about 20.4%. The slope of the evacuation curve also gradually decreased, which is in agreement with the changing trend in the basic evacuation simulation model. Through multiple simulations, it is found that the reason for this phenomenon is that in the early stage of evacuation, people in the studied space were scattered and everyone can choose the nearest exit to escape; subsequently, people closer to the exit first evacuated to the outdoor safety area, while people far from the exit (e.g., at the station level and especially the platform level) was blocked near the stairway entrance. When these people reached the station level through the stairs and then reached the exit, they tended to select the closest exit. The exit far away from them remained idle.

**Fig 6:**
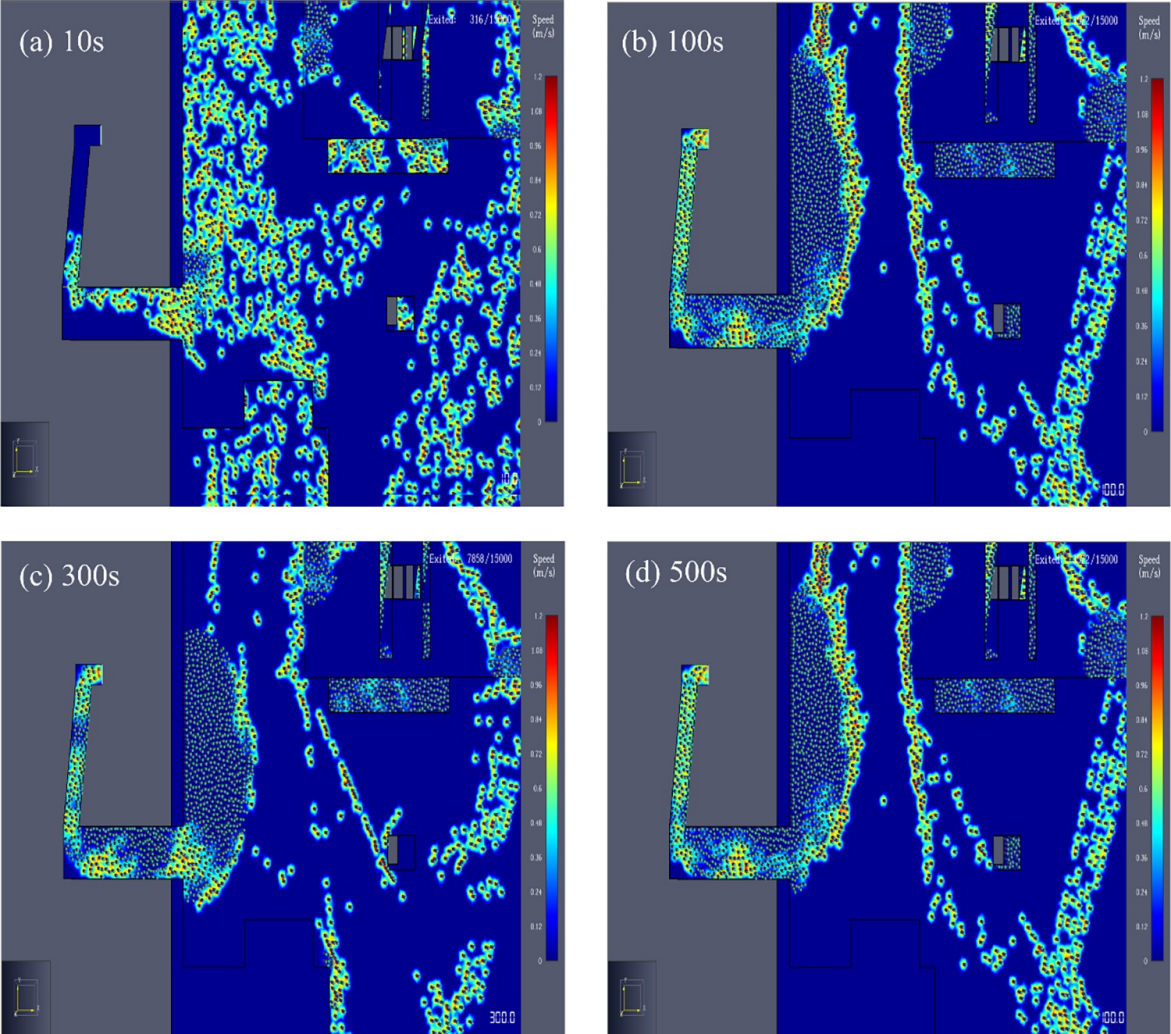
Flow rate and speed distribution of evacuees near Exit B at different moments. (a) 10s, (b) 100s, (c) 300s, and (d) 500s.

**Fig 7:**
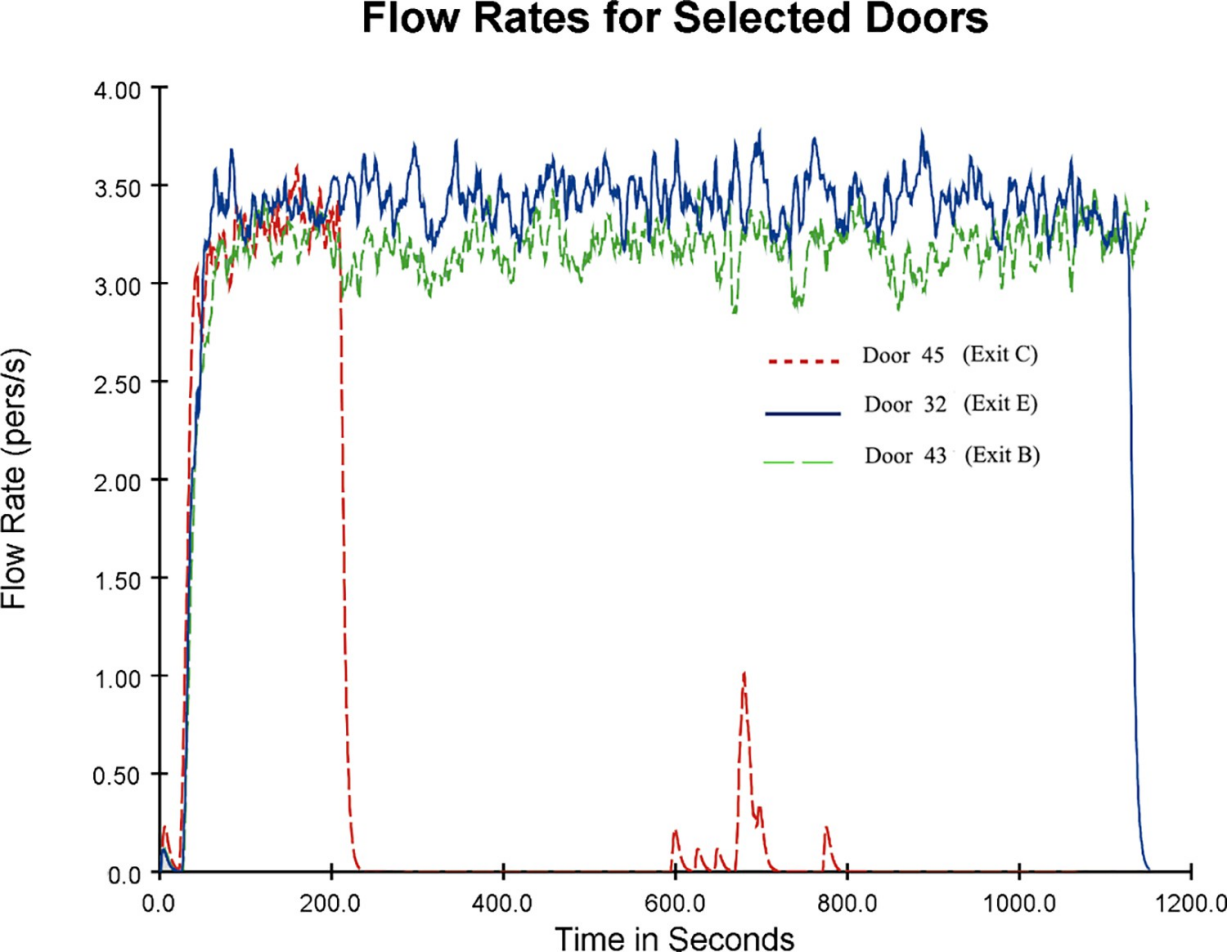
Flow variation diagram for Exits B, C and E (basic evacuation simulation model).

In the basic evacuation simulation model, all exits were fully utilized in the early evacuation stage. As time passed, some exits were idle and no one passed through. People were mainly concentrated in Exits B (Door43), C (Door45) and E (Door32), causing severe congestion at these three exits. Exit B was taken as an example for further analysis. The number and speed distribution of evacuees in the simulated evacuation process with time are shown in Fig 6. The number of evacuees at Exit B at 10 s was small and the speed was low. As evacuees continued to gather at Exit B, many evacuees appeared within 100~500 s. Most evacuees moved at a speed of less than 0.12 m/s or even remained stationary, indicating that Exit B was seriously congested. According to the simulation animation, Exits C and E showed the same situation (not to be enumerated). The flow change diagram of Exits B, C and E is shown in Fig 7.

**Fig 8:**
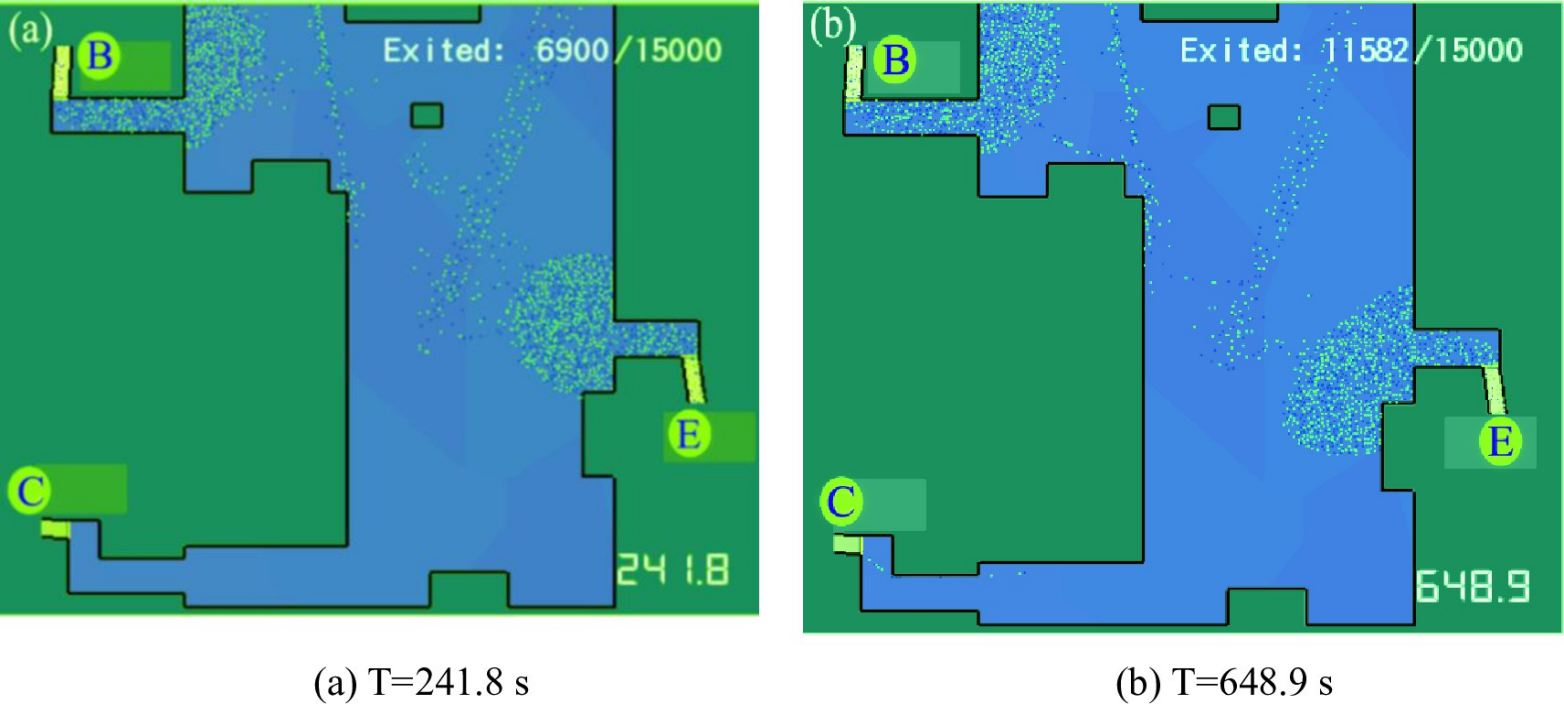
Screenshots of evacuation status at different time points of the 3D evacuation animation. (a) T = 241.8 s and (b) T = 648.9 s.

Combined with the 3D evacuation animation, it is found that the flow rate at Exit C (Door 45) became 0 at 241.8 s, and the flow of people passed through the exit between 593–802 s. Then, the flow rate became 0. Two representative time points (241.8 s and 648.9 s) in the 3D evacuation animation were selected, as shown in Fig 8(a) and 8(b). The flow change during the evacuation at each exit is demonstrated in Fig 7. In other periods, Exit C was idle, while Exits B and E showed more serious congestion. Since Exit C was far away, people were more accustomed to escaping from the exit closer to them. This can also be attributed to the herd behavior of the evacuated crowd, i.e., seeing others queuing at Exits B and E, they follow the people next to them to do the same. However, between 593 and 802 s, some people chose Exit C, which was farther away, indicating that not all people had a herd mentality and would choose the faster exit to escape according to their analysis. This also confirms the results of previous studies on herd mentality during an emergency evacuation, where directional herding and distance herding existed in the evacuation process [57–59]. The emergence of herding evacuation psychology results from the lack of guidance rules, which leads to blind actions or herding behavior of people unfamiliar with the underground environment; the lack of auxiliary evacuation facilities causes more chaotic and disorderly behavior.

**Fig 9:**
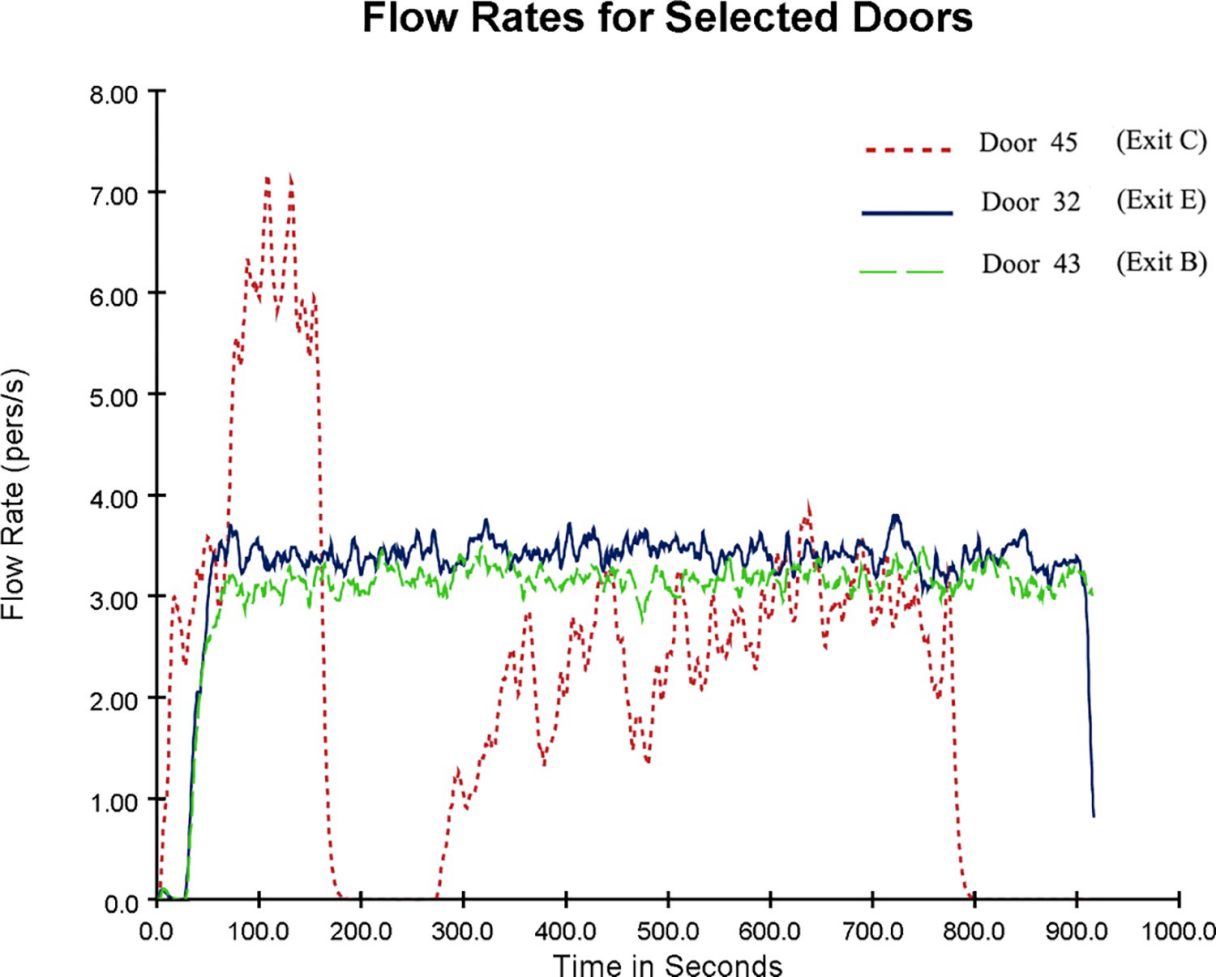
Flow variation diagram for Exits B, C and E (optimized evacuation simulation model).

In the optimized evacuation simulation model, the flow changes at Exits B (Door 43), C (Door 45) and E (Door 32) are shown in Fig 9. Fig 9 shows that the flow rate at Exit C (Door 45) reached a maximum of 7 per/s around 110 s while remaining at 2–3 per/s between 350–800 s. This is mainly due to the effect of the guiding rule. Under the guidance rule, people designated to evacuate from Exit C reached the exit, resulting in the flow of people. This indicates that the utilization of Exit C was improved after the guidance rule occurred.

Through several experimental observations, the specialized routes (equivalent to increasing the width of evacuation exits) and evacuation guidance rules set by the optimized evacuation simulation model reduced the evacuation time. They facilitated evacuation behavior to a certain extent. Increasing the exit width or setting up auxiliary evacuation routes is one of the effective measures to improve evacuation efficiency. However, whether increasing the exit width should be considered according to the actual function and nature of the building. In addition, reasonable evacuation guidance measures are important for the emergency management of complex buildings. There are various evacuation guidance methods [60, 61]. For the emergency management of UUCs, a corresponding reasonable evacuation strategy should also be developed.

**Fig 10:**
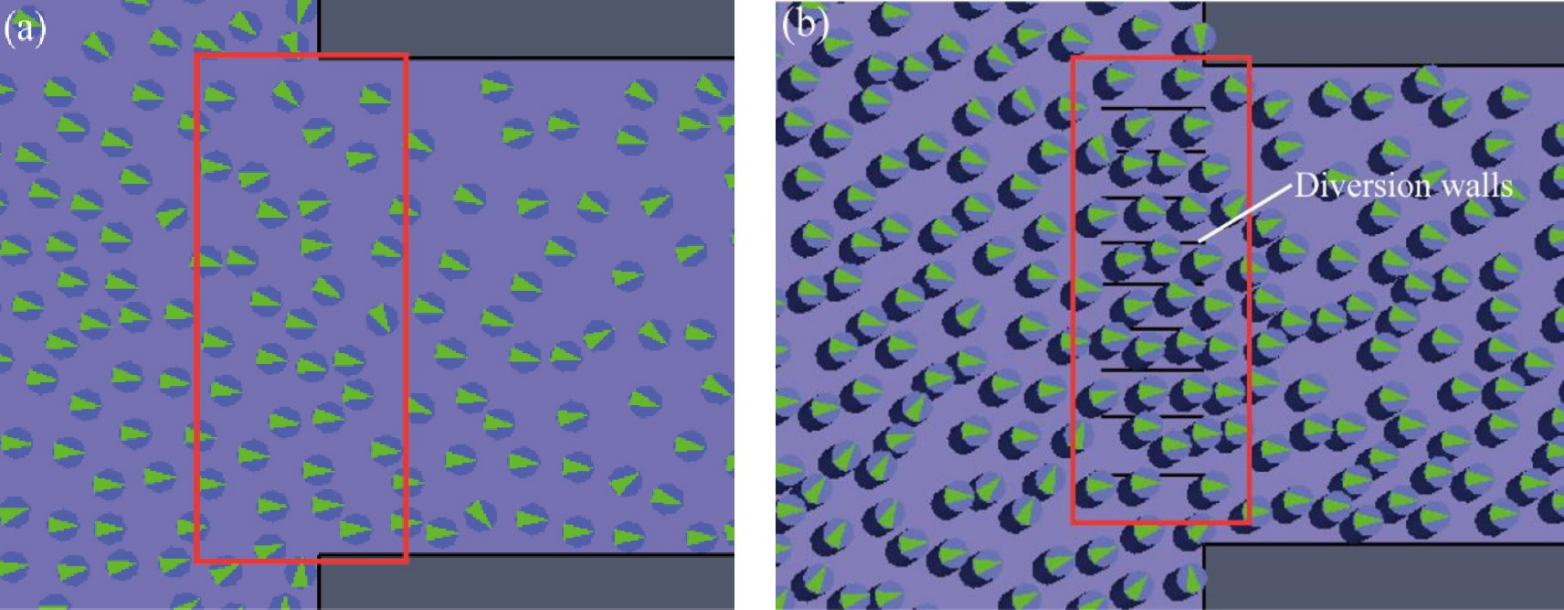
Effects of diversion walls on evacuation effectiveness (a) without diversion walls (basic evacuation simulation model) (b) with diversion walls (optimized evacuation simulation model).

The authors’ previous study has shown that the installation of barriers has a certain diversion effect on evacuation congestion [62]. In the optimized evacuation simulation model, a diversion wall was set up to simulate the evacuation process and compared to the evacuation process without a diversion wall in the basic evacuation simulation model. The influence of the evacuation process with or without a diversion wall was observed, as shown in Fig 10(A) and 10(B). The diversion wall also played a certain diversion role. During the whole evacuation process, the local flow of people near the diversion wall was well organized. People can queue through the exit faster. Due to evacuation, the density of people showed a decreasing trend. Although people behind the diversion wall initially showed a certain arrangement order under certain evacuation guidance rules, people behind the diversion wall showed a more chaotic phenomenon in a certain space during evacuation. This may be related to the conflict between the acute fear psychology during evacuation and the short-term relaxation psychology after the diversion evacuation, which is not conducive to the subsequent evacuation behavior. Therefore, although installing diversion walls in the evacuation process can improve evacuation efficiency under certain circumstances, it may not continually have a positive effect.

## 5. Conclusions

In this study, based on the steering model in Pathfinder, the evacuation process of the Wuhan ‘GG’ underground complex was simulated. Through the comparison and analysis of the basic evacuation simulation model and the optimized evacuation simulation model, the following conclusions are drawn.

1. The locations of stairways, exit gates and supermarket checkout counters in the UUCs were prone to bottleneck effects and caused severe congestion. These locations were critical and should be focused on in the emergency evacuation process.
2. In emergency evacuation in UUCs, increasing the exit width or setting up auxiliary evacuation routes for critical locations prone to evacuation bottlenecks was one of the effective measures to improve evacuation efficiency. In addition, reasonable evacuation rules were a favorable measure for emergency evacuation.
3. During evacuation in UUCs, the directional subordination and distance subordination caused by the evacuation herd mentality affected the emergency management effectiveness due to the unique spatial environment of UUCs and the site environment during emergencies.
4. In the process of emergency evacuation in UUCs, the diversion wall at the exit can improve evacuation efficiency and reduce congestion to a certain extent. However, it may not necessarily produce a positive effect. People showed a more chaotic phenomenon in a certain space behind the diversion wall, which may be related to the conflict between the acute fear psychology during evacuation and the short-term relaxation psychology after the diversion and evacuation.

## Supporting information

S1 Dataset Supporting information A (Fig 5(A)). (CSV)

S2 Dataset Supporting information B (Fig 5(B)). (CSV)

S3 Dataset Supporting information C (Fig 7). (CSV)

S4 Dataset Supporting information D (Fig 9). (CSV)
